# Study protocol: Brief intervention for medication overuse headache - A double-blinded cluster randomised parallel controlled trial in primary care

**DOI:** 10.1186/1471-2377-12-70

**Published:** 2012-08-10

**Authors:** Espen Saxhaug Kristoffersen, Jørund Straand, Jūratė Saltytė Benth, Michael Bjørn Russell, Christofer Lundqvist

**Affiliations:** 1 General Practice Research Unit, Department of General Practice, Institute of Health and Society, University of Oslo, Oslo, Norway; 2 HØKH, Research Centre, Akershus University Hospital, Lørenskog, Norway; 3 Institute of Clinical Medicine Akershus University Hospital, University of Oslo, Nordbyhagen, Norway; 4 Head and Neck Research Group, Research Centre, Akershus University Hospital, Lørenskog, Norway; 5 Department of Neurology, Akershus University Hospital, Nordbyhagen, Norway

**Keywords:** Medication-overuse headache, Chronic headache, Chronic tension-type headache, Migraine, Brief intervention, General practice, Primary care, Cluster randomised controlled trial

## Abstract

**Background:**

Chronic headache (headache ≥ 15 days/month for at least 3 months) affects 2–5% of the general population. Medication overuse contributes to the problem. Medication-overuse headache (MOH) can be identified by using the Severity of Dependence Scale (SDS). A “brief intervention” scheme (BI) has previously been used for detoxification from drug and alcohol overuse in other settings. Short, unstructured, individualised simple information may also be enough to detoxify a large portion of those with MOH. We have adapted the structured (BI) scheme to be used for MOH in primary care.

**Methods/Design:**

A double-blinded cluster randomised parallel controlled trial (RCT) of BI vs. business as usual. Intervention will be performed in primary care by GPs trained in BI. Patients with MOH will be identified through a simple screening questionnaire sent to patients on the GPs lists. The BI method involves an approach for identifying patients with high likelihood of MOH using simple questions about headache frequency and the SDS score. Feedback is given to the individual patient on his/her score and consequences this might have regarding the individual risk of medication overuse contributing to their headache. Finally, advice is given regarding measures to be taken, how the patient should proceed and the possible gains for the patient. The participating patients complete a headache diary and receive a clinical interview and neurological examination by a GP experienced in headache diagnostics three months after the intervention. Primary outcomes are number of headache days and number of medication days per month at 3 months. Secondary outcomes include proportions with 25 and 50% improvement at 3 months and maintenance of improvement and quality of life after 12 months.

**Discussion:**

There is a need for evidence-based and cost-effective strategies for treatment of MOH but so far no consensus has been reached regarding an optimal medication withdrawal method. To our knowledge this is the first RCT of structured non-pharmacological MOH treatment in primary care. Results may hold the potential of offering an instrument for treating MOH patients in the general population by GPs.

**Trial registration:**

ClinicalTrials.gov identifier: NCT01314768

## Background

Headache is a common health problem and the personal burden, social impact and economic cost for both the sufferers and society are substantial [1,2]. Most common headaches are episodic tension-type headache (TTH) and migraine [3]. However, 2–5% of the world’s population have chronic headache [4-13] defined as 15 or more headache days per month for at least 3 months and/or 180 or more headache days per year.

Headache is mostly self-managed [14,15] and headache prescription medications account only partly for the total medication use for headache since most patients buy over-the-counter (OTC) drugs [5,6,16-19]. Headache accounts for 4% of the general practitioners (GPs) consultations [19], and is probably the most common reason for referral to neurologists [19,20]. Approximately 20–30% of all new referrals to out-patients neurological departments are due to headache [21,22].

Analgesic use, misuse and overuse represent major health problems associated with numerous adverse health consequences. A population-based study from Norway which included about 50,000 subjects found that 10% reported taking analgesics currently on a daily basis and up to 5% reported taking analgesics on a daily basis for at least six months [23]. Results from another Norwegian study showed that 28% of men and 13% of women had used analgesics over the preceding 28-day period, mostly to treat headaches [24]. Frequent intake of analgesics may, however, worsen headache and lead to chronification and Medication Overuse Headache (MOH) [25-27]. MOH is a condition with chronic headache in combination with overuse of acute headache medication(s) [25-27]. The prevalence of MOH in the general population is 1–2% [5,6,10,25-28]. The condition was first described for egotamines in 1951 [29] and it is now substantiated that all drugs used for the acute treatment of headache can cause MOH in patients with a pre-existant headache disorder [25-27]. The proportion of MOH is lower in the general population than one sees in clinical settings, and the distribution of the overused medication differs with simple analgesics being most frequently overused in the general population [7,9,17,28,30-35].

The aims of MOH management are [36,37]

i. withdrawal of the overused drug(s)

ii. to provide the patient with pharmacological and non-pharmacological support

iii. to prevent relapse

Detoxification from the overused medication often leads to headache improvement [25,26,31,38], but is often complicated by temporary withdrawal symptoms such as worsening of headache, nausea, vomiting, hypotension, tachycardia, sleep disturbances, restlessness, anxiety and nervousness which typically occur 2–10 days after detoxification [26,27,40,41]. There is no established optimal withdrawal method for MOH though many different strategies have been suggested [25,36,37,39]. These include use of antiemetics and/or neuroleptics to reduce abstinence-like symptoms, intravenous administration of ergotamines and substitution of the offending painkiller with another. Steroid treatment has also been used to alleviate withdrawal reactions though this strategy is controversal [36,37,41-43].

Regarding prophylactic headache medication, there is also an ongoing discussion whether this should be initiated immediately at withdrawal or after completed withdrawal therapy [36,44].

Follow-up studies of various duration have reported relapse rates between 20–60% and findings from these studies suggest that patients have the highest risk of relapse within the first year after withdrawal [37,45-49].

MOH is a heterogenous disorder which has been suggested to include both subgroups with simple medication overuse as well as more complex detoxification-resistant cases [36,50-52]. Some of these cases may be more “dependency-like” and it has indeed been suggested that MOH shares some common neurobiological pathways with drug dependence and that MOH therefore may represents a kind of addictive behaviour [53]. Whether this applies to all MOH cases or specific subgroups defined by this particular “dependency-like” behaviour (eg. “complex” MOH) remains to be demonstrated. Two studies have demonstrated that most MOH patients fulfill criteria for dependency according to the Diagnostic and Statistical Manual of Mental Disorders, fourth edition (DSM-IV) [54-56]. Another study found that the dependency score based on the Leeds Dependency Questionnaire was similarly increased in MOH patients and illegal drug addicts [57].

Over the past decades several dependency assessment scales have been developed. The Severity of Dependence Scale (SDS) is a simple, validated scale which scores psychological dependence on a number of different substances [58-63]. Previous studies from our group have revealed that the SDS has both high sensitivity, specificity, positive and negative predictive values for detecting persons with MOH among chronic headache patients. [30,35,64]. In addition, the SDS score has been shown to predict likelihood of successful detoxification in a general population [65].

Screening and Brief Intervention (BI) is a well-known approach to identify and treat unhealthy alcohol use [66]. The SDS and similar scales such as the Alcohol Use Disorders Identification test, have previously been used to identify individuals at risk for addiction-related problems [58-63,67-69]. BI involves the use of such an identification tool followed by feedback to the identified individual as being “at risk”. The final step, in this very short and simple intervention is to give information suggesting to cut down the use of the particular substance to predecided “acceptable” levels [66]. BI includes clear directive advice, but focus is also on increasing patients insight and awareness regarding overuse as described in more detail elsewhere [66,70]. The BI method has shown promising results with both short- and longlasting reduction of alcohol intake and levels of related biochemical markers such as liver transferase levels [66,71-73]. Similar methods have also been successfully applied for various other addictive drugs [70,74,75].

We have previously reported data from an open, un-controlled study of medication overuse headache in the general population, which suggested that three out of four MOH subjects had managed to reduce their medication intake after short information [76]. Similar simple advice also works in clinic settings [77,78]. One population- and one clinic-based study suggest that MOH can be successfully managed in a primary care setting after an initial collaboration with headache specialists [45,79].

The common headache disorders require no high-tech investigations and may therefore be diagnosed and managed by all skilled physicians. Most headaches are therefore probably best managed in primary care. Focus on MOH in primary care is therefore important both in order to prevent MOH from developing and for early diagnosis and treatment.

We have designed a BI for treatment of MOH in primary care and planned a double-blinded cluster randomized parallel controlled trial (RCT) to evaluate effects of the intervention.

### Objectives

The primary objective is to evaluate the effects of a brief intervention (BI) versus business as usual (BAU) in the management of MOH in primary care.

## Methods/Design

**Figure 1:**
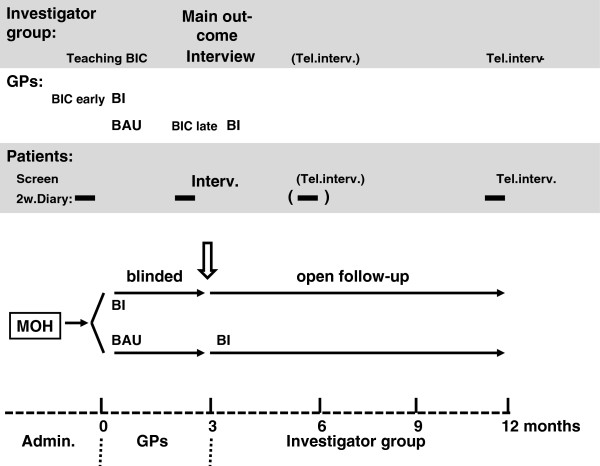
**Flow-chart of study*.*** Figure illustrates main time line with the different phases with those mainly responsible for each phase (lower part). Upper part shows time-points for Patients data collection (2 week headache diaries (2w.diary) and interviews) as well as timing of various moments for the Investigator group and GPs with Brief Intervention training courses (BIC) for GPs and intervention (Brief intervention - BI and Business as usual – BAU respectively). Main outcome time-point at 3 months depicted with double arrow and bold writing. BI, Brief intervention; BIC, Brief Intervention course early or late; BAU, Business as usual; GP, General practitioner; Rand, randomisation.

Our hypothesis is that BI is more effective than BAU. The hypothesis will be tested using a double-blinded cluster randomised parallel controlled clinical trial in primary care comparing BI and BAU three months after study inclusion and with additional open one year follow-up (Figure 1).

### Setting

The Norwegian GP list-patient system was established in 2001. The GPs are reimbursed through a fixed annual fee and fees for the specific services from the National Health Insurance and the patients. GPs act as gate keepers for referrals to secondary care specialists and hospitals except in emergencies. GP specialists must renew their specialty every fifth year. As a part of this process, clinical training courses and participation in peer continuous medical education (CME) group meetings are compulsory. GPs in the same practice often attend the same peer CME group, each group typically comprised of four to ten GPs from different practices. The study will be undertaken among GPs attending CME groups in south-eastern Norway.

### Recruitment, randomization and blinding

#### GPs

Recruitment of the GPs will be done by inviting a number of GPs’ peer CME groups to a Brief Intervention Course (BIC). Randomisation of GPs (and thereby their patients) to either BI or BAU will be performed by an external statistician. Each GP and his/her patients defines one cluster. For practical reasons and to avoid carry-over effects, the CME groups will be the randomisation unit.

GPs in half of the CME groups will receive the BIC and apply BI on their own patients, while the others will run their clinical practice as usual (BAU).

#### Patients

A short validated screening questionnaire for headache [80,81] including questions about headache frequency, intensity (as recorded on a visual analogue scale (VAS) [82]), presence of migraine and medication use will be mailed to all 18–50 year old patients on participating GPs’ patient lists. Names and addresses will be extracted from GP lists using a specially designed software (Mediata Ltd, Tønsberg, Norway). One written reminder will be sent to non-responding patients.

All patients with 15 or more days with self-reported headache per month and headache medication utilization on 10 days or more per month will be invited to participate.

Patients unable to participate in an interview in Norwegian will be excluded.

Baseline patient information (screening questionnaire and headache diary) will be collected by the Akershus University hospital research administration unit before any study-related contact between patient and their GP.

To avoid unblinding and carry-over of information from the BI to BAU, both GPs and patients will initially only receive basic information that this study aims to evaluate headache and headache care in primary care.

After the intervention part, all follow-ups will be conducted by the first author (ESK), who will be blinded in relation to which treatment the participant has received. All participating patients will receive a semi-structured interview and clinical and neurological examination.

#### Population controls

1. Control group; random sample without chronic headache (headache if present must be <15 days per month) based on the screening questionnaire. This group will be used to control for burden of headache in terms of quality of life and costs. It will also be used for drop-out analyses.

2. Chronic headache control group; random sample with headache ≥15 days per month but without medication overuse based on the screening questionnaire. This group will be used as a control for the natural course of chronic headache as well as for drop-out analyses.

### Intervention

#### Brief intervention course (BIC)

The participating GPs will receive a one day course held by headache specialists (CL and ESK). It includes general lectures about migraine, tension-type headache and chronic headaches, especially MOH. A presentation of the Brief Information scheme will be given with practical instructions examplified by role play. Participating GPs will earn CME credits by the Norwegian Medical Association as part of the GP training curriculum. In general, most of these physicians have previously not received specific training in the handling of MOH. However, information on possible previous participation in such training will be collected.

#### Brief intervention (BI)

**Figure 2:**
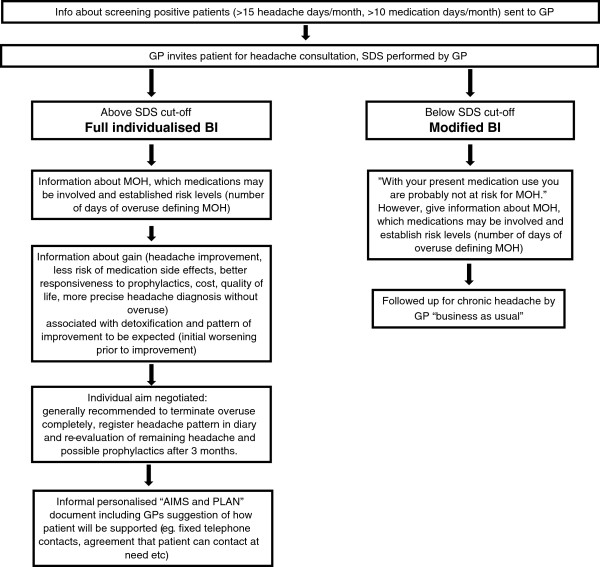
Flow-chart of GPs Brief Intervention for MOH strategy.

GPs allocated to the BIC will receive information about screening-positive patients on their lists. These patients will be invited to a consultation for headache by the GP. BI will then be performed as follows (for more details see flow-chart, Figure 2):

1. Use of the SDS questionnaire (Table 1) to identify patients at risk for MOH, i.e. SDS cut-off values ≥5 (females) and ≥4 (males) [30,35,36]. Inform the patient that he or she is identified as being at risk for MOH. Patients with SDS score under cut-off will also receive a structured intervention and information about the relationship between drug intake and headache and the importance of staying within “safe levels” of medication use.

2. Give short structured information about MOH and the association between medication overuse and chronic headache.

3. Give specific individualised information and advice regarding reduction of acute headache medication.

**Table 1:** The five questions of the Severity Dependence Scale (SDS) adapted for headache medication

1.	Do you think your use of headache medication was out of control? (never/almost never=0, sometimes=1, often=2, always/nearly always=3)
2.	Did the prospect of missing a dose make you anxious or worried? (scoring as for question 1)
3.	Did you worry about your use of your headache medication? (scoring as for question 1)
4.	Did you wish you could stop? (scoring as for question 1)
5.	How difficult would you find it to stop or go without your headache medication? (not difficult=0, quite difficult=1, very difficult=2, impossible=3)

#### Headache classification

The headaches will be classified according to explicit diagnostic criteria of the ICHD-II and it‘s relevant revisions [83-86]. In this study we have defined chronic headache as 15 or more headache days per month for at least 3 months and/or 180 or more headache days per year as the ICHD-II does not provide an explicit definition for all headache diagnoses. The ICHD-II diagnosis will be made by the first author and by consensus in the project team based on the 3 month follow-up interview.

#### Headache diary

A modified version of a validated headache diary [87,88] will be used in order to prospectively record data on headache frequency, headache intensity (VAS) and medication. We have also added self-reported number of sick-leave days. The completion of the diary for a two week period will be required at baseline, at three, six and twelve months for both BI and BAU patients. Written instructions for the completion of the diary will be mailed to the patients.

#### Blinded three months follow-up

Three months after the intervention (or after recruitment to the BAU arm), the patients will be invited to a clinical interview with an examination focusing on diagnosis of headache, relevant comorbidity, use and quantification of prescription drugs as well as OTC drugs. The interviewer will be blinded as to which study arm the patients are allocated to and the patients will be blinded as to intervention, outcomes and aim of study.

Additional questionnaires will be completed by the patients (Table 2) in order to cover headache related health and quality of life, use of health services, socioeconomic and demographic data.

**Table 2:** Validated questionnaires used for additional outcomes with comments regarding relevance for present study and references to the literature list

***Validated questionnaire***	***Description of questionnaire***	***Importance in present study***	***Ref.***
**Short Form-36 (SF-36)**	Most used general QoL questionnaire	Enables quality of life comparison with non-headache patients from the general population ., normal material for Norwegian population exists	[89-91]
**Migraine Disability Assessment (MIDAS) Headache Impact Test – 6 (HIT-6)**	Most used headache specific QoL instruments, recommended for RCTs of headache treatment	Enables comparison between various headache categories	[92,93]
**Severity of Dependence Scale (SDS)**	Validated for detection of MOH in previous studies	Simple identification of MOH patients, comparison against scores for other addictive drugs	[58,94]
**Mini International Neuropsychiatric Interview (MINI)**	Validated structured interview for DSM-IV based diagnosis of substance dependency	Gold standard for dependency for validation of SDS	[58,94]
**Eysenck Personality Questionnaire (EPQ)**	Widely used personality scale both in relation to drug dependency and in more general	Assessment of personality as a factor which may affect outcomes after a behavioural intervention, Norwegian population standards exist	[95-97]
**Hopkins Symptom Checklist-25 (HSCL-25)**	Well validated scale for symptoms of psychological distress	Supplement to Eysenck for assessing degree of distress which may affect outcomes of behavioural intervention, Norwegian population standards exist	[98-100]
**Hospitality and Anxiety Depression Scale (HADs)**	Most used scale in clinical materials for scoring depressive and anxiety symptoms	Assessment of depression and anxiety as comorbidities of chronic headache which may affect outcomes of intervention, Norwegian population standards exist	[101,102]

#### Twelve months follow-up

A re-interview of participants from both study arms done by telephone by the same interviewer will be performed after one year focusing on headache diagnosis, use of medication, and present burden of headache (number of headache days and intensity of headache). Headache associated quality of life will be assessed with MIDAS and HIT-6. SDS will be measured.

### Outcome measures

Outcome measures are listed in Table 3. Primary outcomes are headache days and medication days per month comparing the two arms and change compared to baseline. With a simple, non-medication intervention as the present, any significant improvement in these parameters is judged to be of relevance. Proportions fulfilling commonly used clinical definitions of chronic headache (≥15 days/month) and medication overuse [75-78] at follow up are also clinically relevant and included as secondary outcomes. In addition, since clinically relevant outcomes in medication studies have been suggested to be 25 to 50% improvement in headache days and headache index, these are also included as secondary outcomes. 

**Table 3:** Outcome measures and time points for application of the various outcomes (statistical calculations are further described in the Statistics section)

	**3 months**	**12 months**
**Primary outcomes**		
Difference between BI and BAU in:		
a) number of headache days per month	X	
b) number of headache medication days per month	X	
Change relative to baseline in		
a) number of headache days per month	X	
b) number of headache medication days per month	X	
**Secondary outcomes**		
Difference between BI and BAU in proportion of cases without chronic headache and without medication overuse	X	
Change relative to baseline in proportion of patients with more than		
a) 25% reduction of headache days	X	X
b) 50% reduction of headache days	X	X
Change relative to baseline in headache index (Area under curve for headache intensity versus time)	X	X
Change relative to baseline in		
a) headache days from headache diary	X	X
b) medication days from diary	X	X
Change relative to baseline in average headache intensity recorded by VAS (from headache diary)	X	X
Change relative to 3 month follow-up in self-reported health related costs		X
Change relative to 3 month follow-up in quality of life recorded as SF-36 and MIDAS/HIT-6		X
Change relative to baseline in:		
a) number of headache days per month		X
b) number of headache medication days per month		X
Relapse rate compared with status at 3 month		X

*Abbreviations: BI* brief intervention, *BAU* business as usual, *VAS* visual analogue scale, *SF-36* short form-36, *MIDAS* migraine disability assessment score, *HIT-6* headache impact test-6.

### Data handling and statistical analyses

#### Power calculations

According to the Norwegian Medical Association, the average number of listed patients pr GP is approximately 1200. Using 1000 patient pr GP (simplicity) gives us (based on previous studies [4,5]) an estimate of approximately 30–40 patients with chronic headaches and 10 patients with MOH per GP.

Using previous results from studies from Akershus University Hospital [76] regarding number of days of medication intake per month and proportion of patients with headache more than 15 days per month before and after unstructured information about MOH, we have made an approximation of the required sample size.

Using 80% power for the detection of a similar sized difference as found in the previous study for number of days of medication intake [76], we would need 18 patients in each arm unadjusted and five clusters (ie. GPs) with eight patients each, to achieve significant results at the 5% level. For analysis of the proportion of patients with more than 15 days of headache, power calculations yield 30 patients or eight clusters (GPs) with eight patients each. Since the intra-cluster coefficient of correlation is not known (here estimated at 0.5 as a “worst case”) and since we don’t know the degree of carry-over of information from one group of GPs to another, we assume a sample size of 20 physicians (160 patients) to be reasonably safe. This would also give significant results at the 5% level even if only five patients per GP were included.

#### Statistics

All analyses will be focused at the patient level (inference unit will be patient). We will perform a series of frequency analyses tabulating outcome variables against various explanatory variables and/or confounders. Suitable descriptive statistics will be used. Since individual observations within the same cluster (ie. patients of one GP) may be correlated, the intra-cluster (intra-class) correlation coefficient (ICC) measuring such a “clustering effect” and estimating the relative variability within and between clusters will be calculated for both intervention- and control groups. Analysis of differences between BI- and BAU groups based on mixed linear models (MLM) will be performed to account for the hierarchical nature of data, where the main point is to allow variation to be modelled at each level of the data, for example, the GP and the patient level. 95% confidence intervals will be used.

Analysis based on prespecified hypotheses regarding the two primary and the secondary outcomes will be performed as hypothesis testing on the entire dataset and Bonferroni corrections will be used for multiple comparisons. For evaluation of possible outcome predictors where no prior data from this population exist, split file analysis [103] will be used. The splitting of the data set will be performed prior to any analyses of primary and/or secondary outcomes.

The intention-to-treat principle will be followed by including all patients with at least one follow-up response. Missing values will be handled using multiple imputation techniques.

SPSS 16.0 and SAS will be used for statistical analyses.

Registration of electronic data from the semi-structured interviews will be done by using Snap Survey (Snap Survey, London, UK).

The participating patients will receive a weekly reminder regarding the headache diary via mobile phone (SMS) during each two weeks diary period at three, six and twelve months, respectively.

### Ethics and data security

The study has been approved by the Regional Committee for Medical Research Ethics, the Norwegian Social Science Data Services (NSD) and the Norwegian Directorate for Health.

All data will be anonymised. All participating patients and GPs must give informed, written consent. The approval of the Regional Committee for Medical Research Ethics was given based on a possibility of a cross-over from the control group to the intervention group if the main outcomes at three months show a significant beneficial effect of BI. This is to avoid a six months delay in offering effective treatment. In that case, the GPs in the BAU arm will receive the BIC and be able to perform BI on their own MOH patients.

### Pilot study

The intervention has been be tested for practicability and acceptability in a pilot study with six GPs. The pilot study did not involve a control group. Recruitment methodology and logistics were tested. Patients from the pilot study will not be included in the main study.

### Dissemination/feedback of results

After the study, feedback will be given to the involved GPs regarding the efficacy of BI and main outcomes. In addition, we aim to publish the results in international peer-reviewed scientific journals and disseminate our experiences in national medical fora.

## Discussion

There is no consensus for MOH withdrawal programmes and there is therefore a need for evidence-based and cost-effective strategies for MOH [36,37].

To our knowledge, this is the first double-blinded cluster-randomized controlled clinical trial for MOH in primary care.

The GPs that will be included in the present study are assumed to be representative for Norwegian GPs in general in terms of localisation (urban vs. suburban), gender and age distribution. Through the Norwegian GP list-patient system, all citizens are listed with a GP. Therefore, with a representative selection of GPs, the patient population is assumed to be reasonably population-based which will increase the external validity of our findings.

The age range of patients (18–50 years of age) has been chosen in order to target the highest number of patients with chronic headache, as the prevalence is lower in younger people and older people have a higher frequency of co-morbidities. We have chosen an upper age limit of 50 years since data from the Norwegian prescription database (NorPD) also indicate that there is an increase in the number and dosage of various relevant drugs (notably anti-hypertensives and cardiovascular drugs) at approximately 50 years of age [104] and we want to monitor headache medication and not the use of drugs prescribed for other illnesses.

Selection bias may occur by just including patients who are willing to cooperate in the intervention; however the same selection will take place in the control group. The selection of participating GPs are based on voluntary participation and the BIC will give them CME credits. Our GPs might therefore have a higher motivation for the BI than GPs in general, but this selection will also take place in the control group. Gains for participating GPs are: i) improvement in headache diagnostics and management ii) receiving a tool for identifying and detoxifying MOH patients.

The study group will cover the normal fee for the BI consultation for the patients, apart from this there are no economic incentives for participation either for the patients or the GPs.

Although questionnaires cannot replace an encounter with a skilled physician, single questions about migraine and tension-type headache and frequency of tension-type headache have been shown to be valid [80,81]. However, especially among those with chronic or co-occurring headache types, diagnosing headaches is not always easy. The gold standard for making a specific headache diagnosis is an interview combined with a physical and neurological examination by a physician experienced in headache diagnostics. All patients will be diagnosed based on interviews by a GP trained and experienced in headache diagnostics also in order to avoid inter-observer variation (ESK).

Some of our data will be based on retrospective self-reports and therefore open to recall bias, although here is no reason to suspect systematic bias. In order to counteract this, we will also use headache data from prospective headache diaries [87]. Recent studies using a similar headache diary with written instructions before first consultation found high usefulness, acceptability and comprehensibility of the diary as well as good compliance and completeness of data [105,106]. We will use the headache diary for periods of two weeks. This period may seem too short for infrequent forms of headache, but in our sample of chronic sufferers this should not be a problem. In addition, a longer headache registration period may reduce compliance and cause greater interference with the cohort.

A common finding in many brief intervention studies and RCTs on alcohol use, are small reductions in alcohol consumption at follow-up also in the control group [73,107]. Possible reasons for this may be motivational effects of screening, sensitization to screening/measurements/follow-up, non-intended advice also in BAU group, and “regression towards the mean”. In this study we have tried to minimize most assessment effects by doing a double-blinded parallel RCT, and the screening questionnaire about medication use for headache is embedded with other questions about headache in the very short screening questionnaire. The control group will not receive the SDS screening before the three months follow-up and will therefore not be affected by this.

The SDS has not been validated against other measurements of dependency in MOH sufferers and, indeed, there is still much discussion as to whether MOH represents dependency [50-55,57]. Being fully aware of this, we will use the SDS score, not as an attempt to define dependency, but rather to distinguish between chronic headache subjects with and without medication overuse [30,35].

A Danish study has shown that feasibility, acceptability and implementation of screening and brief intervention programs for alcohol overuse in primary care may cause more problems than they solve for some GPs because it might be problematic to incorporate a brief intervention and follow-up in a busy daily practice where many other different problems are targeted [108]. It is clear that if physicians, and especially GPs, are to deliver interventions in a busy daily and routine practice, it is of great importance that the interventions are feasible and considered clinically relevant. We have used knowledge and experience from a study on the epidemiology of headache in Norway [5,6], a prescription peer academic detailing study [109,110], and a pilot study to investigate some of these aspects, and used this to design the final structure and contents of both the BIC and the study to be both acceptable for patients and feasible for GPs in daily practice.

To reduce the workload for participating GPs as well as for blinding purposes, the first universal/opportunistic screening for chronic headache and probable medication overuse will be performed by the external project administration prior to the BI. Through this screening we also expect to reach possible chronic headache patients who might not be known as such by their GP. Apart from this initial screening, the study is a pragmatic trial tailored to fit into a busy situation of an everyday GP.

We suggest that strengths of this study include the design and approach with randomly assigned intervention and control groups in accordance with the CONSORT statement for RCTs [111].

The present project holds potentials for making a change in the focus on MOH treatment in particular, as well as the medication use for chronic headache in general. The dissemination of the results and of the BI methods to inform the Norwegian health care system will be possible because of the primary care strategy. There is a potential of reducing the suffering of MOH in a large, but so far largely neglected group of patients. Effective treatment at the GP-level, is also an advantage if it does not lead to more referrals to specialists. The principle of treatment at the lowest effective level of care, in this case in primary care, is a stated aim in the Norwegian health care system where also the geographical situation often reduces the accessibility to secondary health care services. In addition, a reduction in medication costs, improved headache status, and reduced secondary headache related costs, may lead to economical savings for society as well as benefits for the individual patients. Such effects may potentially be augmented by a greater awareness of GPs, pharmacists and society in general regarding dangers associated with indiscriminate use of pain killers for frequent headache.

## Competing interests

The authors declare that they have no competing interests.

## Authors’ contributions

CL had the original idea for the study and together with JS, MBR and ESK planned the overall design. ESK prepared the initial draft of the study protocol and was the main author of the present manuscript and together with CL carried out the pilot study. MBR supported in the design of the protocol and with scientific input regarding headache. JŠB planned the statistics methodology and was involved in the experimental design. All authors have read, revised and approved the final manuscript.

## Pre-publication history

The pre-publication history for this paper can be accessed here:

http://www.biomedcentral.com/1471-2377/12/70/prepub
